# Algorithm-based advice taking and clinical judgement: impact of advice distance and algorithm information

**DOI:** 10.1186/s41235-022-00421-6

**Published:** 2022-07-27

**Authors:** Bence Pálfi, Kavleen Arora, Olga Kostopoulou

**Affiliations:** Department of Surgery and Cancer, Imperial College London, London, UK

## Abstract

Evidence-based algorithms can improve both lay and professional judgements and decisions, yet they remain underutilised. Research on advice taking established that humans tend to discount advice—especially when it contradicts their own judgement (“egocentric advice discounting”)—but this can be mitigated by knowledge about the advisor’s past performance. Advice discounting has typically been investigated using tasks with outcomes of low importance (e.g. general knowledge questions) and students as participants. Using the judge-advisor framework, we tested whether the principles of advice discounting apply in the clinical domain. We used realistic patient scenarios, algorithmic advice from a validated cancer risk calculator, and general practitioners (GPs) as participants. GPs could update their risk estimates after receiving algorithmic advice. Half of them received information about the algorithm’s derivation, validation, and accuracy. We measured weight of advice and found that, on average, GPs weighed their estimates and the algorithm equally—but not always: they retained their initial estimates 29% of the time, and fully updated them 27% of the time. Updating did not depend on whether GPs were informed about the algorithm. We found a weak negative quadratic relationship between estimate updating and advice distance: although GPs integrate algorithmic advice on average, they may somewhat discount it, if it is very different from their own estimate. These results present a more complex picture than simple egocentric discounting of advice. They cast a more optimistic view of advice taking, where experts weigh algorithmic advice and their own judgement equally and move towards the advice even when it contradicts their own initial estimates.

**Supplementary Information:**

The online version contains supplementary material available at 10.1186/s41235-022-00421-6.

## Significance statement

Research on advice taking has consistently found that people discount advice from other people and from algorithms alike, especially when it contradicts their own judgement. This “egocentric advice discounting” may undermine the potential of algorithmic tools that are developed to support judgements and decisions. Nonetheless, previous studies employed tasks with outcomes of low importance, they typically recruited students, and they rarely used real advice. Therefore, this study aimed to investigate whether the phenomena of the advice taking literature can be replicated in the clinical domain using realistic vignettes of hypothetical cancer patients, algorithmic advice from an existing, validated cancer risk calculator, and general practitioners (GPs) as participants. The main findings of our study are that GPs, on average, weighed their own judgements and the algorithm equally, and that the influence of advice did not diminish substantially when a conflict emerged between a GP’s judgement and the algorithm. These results challenge the generalisability of the egocentric advice discounting model, and they cast a more optimistic view of advice taking suggesting that algorithmic advice carries great potential to influence clinical judgements.

## Introduction

Evidence-based statistical models and formulas, hereafter algorithms, are produced in ever-increasing number to support the judgements and decisions of lay people and experts alike (e.g. Logg et al., 2019). The idea that algorithms could guide or even replace human judgement is not new. Algorithms predicting patient behaviour were shown to outperform the predictions of psychologists several decades ago (Meehl, 1954). A meta-analysis confirmed that this finding generalises beyond mental health and can be demonstrated in a variety of fields from general medicine to education and business forecasting (Grove et al., 2000). The benefits of using algorithms can be especially high in medical diagnosis, and, in particular, in cancer diagnosis, which accounts for a substantial share of diagnostic errors (Gandhi et al, 2006). The early diagnosis of cancer can be difficult even for experienced physicians as the presenting symptoms can be vague and easily attributed to other, more common conditions, while a delayed diagnosis can increase patient morbidity and mortality and impose heavy costs on the health systems (Neal, 2009). To improve the early diagnosis of cancer in the UK, evidence-based cancer risk calculators were incorporated into the software systems of general practitioners (GPs). For instance, QCancer offers advice in the form of probability estimates (Hippisley-Cox & Coupland, 2013a, 2013b). By taking into account patient demographics, risk factors and presenting symptoms, the algorithm can estimate the probability of the patient having a cancer.

Even though the potential of these algorithms to improve health outcomes is high, no experimental research has been done to understand how GPs utilise advice from algorithms. In the present study, we aim to explore the extent to which GPs’ judgements are influenced by algorithmic advice from a cancer risk calculator, and the factors that may moderate this influence. The potential benefit of this experimental study is twofold. First, understanding how GPs use algorithmic advice can underlie recommendations for introducing them to clinical practice. Second, investigating this question in an experimental setting, using an ecologically valid estimation task with outcomes of high importance and experts as study participants, provides a unique opportunity to test the generalisability of the phenomena and theories of the advice taking literature.

### Advice discounting

Research in the field of advice taking has demonstrated that humans can successfully utilise advice to improve the quality of their judgements and decisions. This has been found in many experiments using a range of quantitative estimation tasks from general knowledge and cue learning to business forecasting (e.g. Goodwin & Fildes, 1999; Harvey & Fischer, 1997; Hütter & Fiedler, 2019; Logg et al., 2019; Önkal et al., 2009; Soll & Larrick, 2009; Yaniv, 2004a, 2004b; Yaniv & Kleinberger, 2000). Nonetheless, advice is rarely used to its full potential because people typically do not take sufficient account of it. They overweigh their own estimation relative to that of an advisor, a phenomenon known as ‘egocentric advice discounting’ or simply ‘advice discounting’ (Yaniv & Kleinberger, 2000; Yaniv, 2004a, 2004b).

Advice discounting has been attributed to multiple mechanisms, which are not mutually exclusive. One such mechanism is anchoring: people tend to anchor too closely to their initial estimate, which reduces deliberation on the advice and prevents the sufficient adjustment of the initial estimate (Lim & O'Connor, 1995; Tversky & Kahneman, 1974). Another hypothesised mechanism is information asymmetry between the judge and the advisor: the judge has access to her own justifications but she is not aware of the justifications of the advisor (Yaniv & Kleinberger, 2000; Yaniv, 2004a, 2004b). This account draws from Support theory which purports that the judged probability of an uncertain event is a function of how detailed the description of that event is (Tversky & Koehler, 1994). Following the information asymmetry hypothesis, improved transparency about the justifications of the advisor should increase the judge’s reliance on the advice. Finally, overconfidence may also contribute to advice discounting: overconfidence in one’s own abilities and beliefs can prevent one from seriously considering the advice of others (Block & Harper, 1991; Kruger, 1999).

Advice taking is generally investigated in the judge-advisor system: study participants (“judges”) are asked to estimate an uncertain quantity; they are then provided with a piece of advice from a defined source (the “advisor”) and, finally, they have the opportunity to update their initial estimates in light of the advice (Sniezek & Buckley, 1995). This research paradigm allows the quantification of the advice’s impact on the final judgement. This impact can be expressed either in absolute values, by simply calculating the difference between final and initial estimates, or in relative values, by taking into account the distance between the initial estimate and the advice. This relative value is the index called “weight of advice”, which represents the percentage shift towards the advice (WoA, Harvey & Fischer, 1997). WoA can take values between 0 and 1, with 0 denoting no judgement updating (initial and final estimates are identical), and 1 denoting full updating (initial estimate equals the advice). WoA scores lower than 0.5 suggest that the final estimate is closer to the initial estimate than the advice and they are typically interpreted as egocentric advice discounting (Yaniv & Kleinberger, 2000). This threshold is crucial for another reason: judgement accuracy could be maximised by averaging the initial estimate and the advice, if the advisor was randomly selected from the same population as the judge (Dawes & Corrigan, 1974; Einhorn et al., 1977). Hence, advice discounting is tightly associated with unrealised performance gains (e.g. Logg et al., 2019).

The first study ever to report a WoA index used a task that combined estimation with cue learning. Study participants (“judges”) were asked to estimate the number of infected cattle in Britain based on two cues: (1) the extent of the covered area presented on a map of the country, (2) the colour of the covered area suggesting the dangerousness of the virus (Harvey & Fischer, 1997). The judges had a short learning period during which they received feedback with the correct estimates, so that they could calibrate their own estimates. After the learning period, no more correct feedback was provided; instead, judges were provided with advice that was presented as coming from other people who performed the same task. Across three experiments and multiple experimental conditions, WoA mostly remained between 0.2 and 0.3. These scores indicated that judges preferred their own estimates over the advice and only adjusted them by a token amount. Later studies using human advice produced consistent findings. WoA scores substantially lower than 0.5 have been reported in general knowledge tasks (e.g. Hütter & Ache, 2016; Hütter & Fiedler, 2019; Soll & Larrick, 2009; Yaniv, 2004a, 2004b; Yaniv & Kleinberger, 2000), business or geopolitical forecasting tasks (e.g. Logg et al., 2019; Önkal et al., 2009), and a wide range of perceptual judgement tasks (e.g. Gino & Schweitzer, 2008; Gino et al., 2012; Logg et al., 2019). These studies usually included multiple experiments, where the characteristics of the advisor or the advice were manipulated. However, WoA rarely approached 0.5, suggesting that discounting of human advice is a robust phenomenon.

Studies using algorithmic advice have also demonstrated advice discounting. These studies can be assigned to three groups based on the information provided to participants about how the algorithm produced the advice: (1) no information, (2) advice was calculated from past human judgements and (3) advice was based on an evidence-based statistical model. Many studies in the first two groups showed evidence of moderate willingness to follow algorithmic advice. For instance, WoA scores ranged between 0.35 and 0.45 in a series of perceptual and forecasting tasks (Logg et al., 2019). Moreover, WoA was 0.41 in a general-knowledge task (Hütter & Fiedler, 2019). Nevertheless, when Logg et al. (2019) recruited national security professionals for one of their experiments that included multiple tasks, they found only a token amount of estimate updating (WoA scores between 0.15 and 0.30) even in tasks unrelated to the participants’ expertise (e.g. business forecasting).

The WoA scores of studies using evidence-based algorithms also paint a heterogenous picture. Low estimate updating was found in a business forecasting task (WoA = 0.28; Önkal et al., 2009) and an associative learning task (WoA = 0.25^1^; Gardner & Berry, 1995) even though both studies explicitly communicated to participants that the algorithmic advice was of good quality. In the associative learning task, participants were non-medical students with no prior experience of the task. They were asked to maintain the vital signs (e.g. blood pressure and heart rate) of hypothetical intensive care patients within acceptable boundaries by estimating the doses of drugs that needed to be administered. They were not informed about the strength of the association between drugs and vital signs. However, they were provided with feedback and could monitor how the patients’ vital signs changed in response to the estimated medication dose. In other words, they were provided with high quality advice that they could verify. Nevertheless, they heavily discounted the advice when making their final estimates. In contrast, a study providing advice from an established algorithm in a business forecasting task found that judges put roughly equal weights on their own estimates and the advice, slightly favouring their own estimates (Lim & O'Connor, 1995).

In sum, most empirical research on advice taking suggests that advice coming from either humans or algorithms tends to be discounted, resulting in suboptimal performance. However, the generalisability of these findings can be challenged. Most of the above studies employed tasks with outcomes of low importance (e.g. general knowledge questions, perceptual judgements). Furthermore, the typical study participants were students who had no/limited prior experience of the tasks. It is therefore unclear whether these findings can be generalised to experts performing real life tasks with important implications, such as GPs estimating a patient’s risk of cancer with the help of a cancer risk algorithm that is based on facts rather than human judgement.

### Advice distance effect

Past research has focused on understanding how the distance between the initial estimate and the advice (advice distance) influences advice utilisation (e.g. Yaniv, 2004a, 2004b). This cue is likely to be crucial in the context of algorithmic advice use by clinicians. For example, a qualitative study about the usage of an established cancer risk algorithm, where 15 GPs consulted with standardised patients (actors), found that experienced GPs claimed to ignore algorithmic advice when it conflicted with their own judgement (Chiang et al., 2015). Although the study did not actually measure to what extent the algorithm was integrated with clinical judgement, the distance between the algorithm and clinicians’ intuitive estimates is clearly an important area of investigation, given the proliferation of algorithmic tools in medicine.

Early research in the advice taking literature concurred that there is a negative linear relationship between advice distance and advice weighting (Minson et al., 2011; Yaniv, 2004a, 2004b; Yaniv & Milyavisky, 2007). This finding is in line with the predictions of social judgement theory (Sherif & Hovland, 1961) that aims to explain the dynamics of attitude change brought about by influential messages. According to this theory, attitude change declines as the distance between the message and the initial attitude increases, because people tend to reject opinions extreme to their own, which prevents the reconsideration of their initial position. When change is considered in relative values (e.g. WoA), the predicted relationship is a negative linear trend. When change is measured in absolute values, the theory predicts a reversed U-shape relationship between attitude change and attitude distance (note that large attitude change is unlikely when distance is small, as it would result in a larger change than intended by the influencing message). Indeed, many studies found evidence for a strong quadratic trend between attitude change and attitude distance (e.g. Freedman, 1964; Insko et al., 1966).

Building on social judgement theory, the advice distance effect was interpreted as a sign of egocentric advice discounting (Yaniv & Milyavsky, 2007). However, this interpretation was challenged on multiple grounds by Schultze et al. (2015). First, all previous studies used a categorical variable to test the effect of advice distance on estimate updating. For instance, Yaniv (2004b) categorised advice distance as near, intermediate, or far, while Yaniv and Milyavsky (2007) classed it as either near or far. Hence, the independent variable might not have been detailed enough to detect a trend other than the negative linear relationship predicted by egocentric advice taking (e.g. any other form of a negative monotone relationship). A similar issue arises when the dependent variable, estimate updating, is categorical (e.g. Minson et al., 2011). Second, an alternative, stimulus–response model, where stimulus intensity equates advice distance, can also account for the monotone negative relationship between WoA and advice distance. This simple model is rooted in two assumptions: (1) absolute update towards advice is proportional to the intensity of the stimulus (advice distance), (2) sensitivity to stimulus intensity diminishes (Stevens, 1957). Crucially, when estimate updating is expressed in absolute values, the predictions of the two models diverge. The egocentric advice discounting model expects judges to update less when presented with very distant advice than when presented with moderately distant advice. In contrast, the stimulus–response model expects judges to increase the extent of updating as advice distance grows, while only allowing for a deceleration in this increment. That is, the former predicts a strong negative quadratic trend resulting in a reversed U-shape relationship, whereas the latter is consistent with both a positive linear and a weak negative quadratic relationship.

### Information about the advisor

Research on the influence of human advice identified various cues that judges regularly employ to appraise the quality of advice (Yaniv, 2004a). From these cues, past performance and expertise are likely to play an important role in algorithmic advice taking.^2^ The significance of past performance on advice utilisation was demonstrated by several studies in which judges could only assess the performance of the advisors via the constant outcome feedback they received during an estimation task (i.e. judges were not informed explicitly about the past performance of the advisor). These studies consistently found a positive relationship between the general accuracy of advisors and the extent to which judges incorporated advice in their final judgements (Sniezek & Van Swol, 2001; Van Swol & Sniezek, 2005; Yaniv & Kleinberger, 2000). The impact of past performance on advice utilisation is rather strong: when participants in a general knowledge task were randomly paired with either a poor or a good advisor, the average WoA score was 0.26 in the poor advice group, which is comparable to that of a typical advice taking study. In contrast, in the good advice group, the average WoA score was 0.52, implying that judges put more weight on the advice than on their own initial estimate (Yaniv & Kleinberger, 2000). Perceived expertise or credibility of the advisor also positively influences advice utilisation (e.g. Birnbaum & Stegner, 1979; Harvey & Fischer, 1997; Sniezek et al., 2004; Yaniv & Kleinberger, 2000). Importantly, the effect of perceived expertise is independent of the effect of past performance. For instance, in a cue learning task, judges relied on the advice of more experienced advisors more than the advice of less experienced advisors, even though the received advice was identical (Harvey & Fischer, 1997).

### The present research

The purpose of this study is to explore the influence of a cancer risk algorithm on GPs’ risk estimates. We will investigate whether the advice discounting phenomenon can be replicated in the clinical domain using realistic vignettes of hypothetical cancer patients and algorithmic advice from an existing, validated cancer risk calculator (*Question 1*). We also aim to test whether advice taking depends on the distance between algorithmic estimates and GP estimates (i.e. advice distance effect), and which model, egocentric advice discounting or stimulus–response, accounts better for the data (*Question 2*). Our last primary question concerns whether the influence of the algorithm depends on providing information about the derivation and accuracy of the algorithm—similar to the expertise and past performance factors studied in previous studies (*Question 3*).

A secondary question of interest concerns the relationship between confidence and advice discounting. Confidence in one’s own estimates has been found to be negatively associated with advice utilisation (Gino et al., 2012; Harvey & Fischer, 1997; Hutter & Fiedler, 2019; See et al., 2011). To test this relationship, we will measure uncertainty in risk estimates by eliciting confidence intervals around these estimates (*Question 4;* e.g. Yaniv & Foster, 1995, 1997).^3^ We preregistered the hypotheses, the design and the analyses of the study at https://osf.io/d7vsa. NB. A separate publication that analysed different portions of the data answers different preregistered research questions, which are not reported in this manuscript (Kostopoulou et al., 2022).

## Method

### Sample size and recruitment

We powered the study to answer two preregistered research questions (*Questions 2* and *3*). Based on the power analysis, we aimed to recruit at least 116 GPs. For details of the power analysis, see Kostopoulou et al. (2022).

We sent an invitation email to the 400 GPs in our database. They had participated in previous studies by the research group and had given permission to be contacted for participation in future studies. The invitation e-mail included a brief description of the study and a link to an online expression-of-interest form. Interested GPs could sign up to the study by completing this form, where they indicated their NHS e-mail address and unique practice code.

### Materials

#### The vignettes

We prepared 23 vignettes describing hypothetical patients presenting to the GP with one or more symptoms that could be suggestive of colorectal cancer. Most of the vignettes had been developed and used in a previous study (Kostopoulou et al., 2020), and we also constructed new vignettes. The vignettes were designed using QCancer (https://qcancer.org), which calculates the risk of cancer based on a patient’s risk factors, demographics and symptoms (Hippisley-Cox et al., 2013a, 2013b). Information included in the vignettes was limited to factors that can contribute to colorectal cancer according to QCancer to ensure the comparability of the algorithmic risk scores and the estimated risk of GPs. To achieve a sufficient level of standardisation, we used the same structure for each vignette. In each vignette, information regarding the demographics and the risk factors (except family history of gastrointestinal cancer) was presented as a list. Information about the symptom(s) (and the family history of gastrointestinal cancer if appropriate) was displayed as a narrative in a text format (see Fig. 2 for an example vignette). Although the narrative structure created a small level of variation regarding the length of the vignettes, it also helped the vignettes to better approximate the clinical setting and improve the simulation of clinical decision making (Evans et al., 2015). To increase engagement with the vignettes, the narrative section typically started with a brief sentence including one piece of contextual information about the patient`s visit (e.g. who accompanied the patient). Across the 23 vignettes, the colorectal cancer risk ranged from 0.58% to 57.23%, with a median of 4.18% (*M* = 14.10%, SD = 18.97%). There were eight vignettes of low risk (< 3%), four vignettes of moderate risk (> 3% and < 6%) and 8 vignettes of high risk (> 6%). All the vignettes and their associated risk scores can be found in the Supplementary material of the online version of the paper by Kostopoulou et al., (2022) (https://www.nature.com/articles/s43856-021-00069-1).

### Design and procedure

**Fig. 1:**
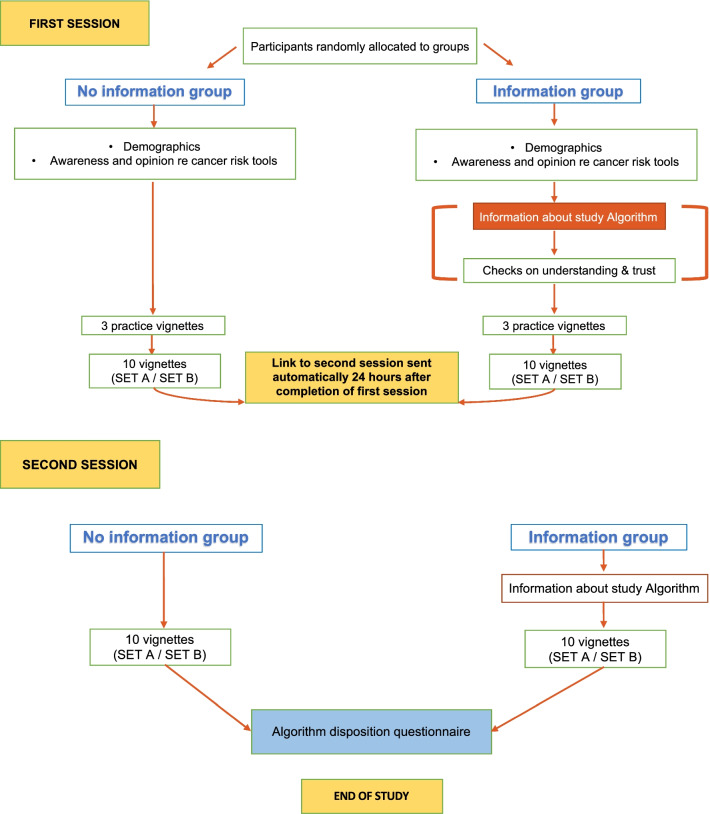
Study flowchart. Figure available under a CC-BY4.0 license at https://osf.io/t7gye/

The study followed a factorial design with one between-groups factor (algorithm information). Figure 1 presents a study flowchart. Participants were randomly assigned to two groups. Half of the participants were provided with information about the algorithm and the other half with no information. Information about the algorithm was presented before participants responded to any of the vignettes. It described how the algorithm was derived, what its estimates meant, and how accurate it was. Specifically, the algorithm description stated:

The algorithm aims to be used as a decision aid, to support 2WW cancer referral decisions. It is not intended to determine those decisions. The algorithm was derived from a large cohort study of 2.5 million patients in the UK. They used data in the primary care record of cancer patients to estimate associations between risk factors, symptoms/signs and a subsequent cancer diagnosis. The algorithm estimates the probability that a patient has colorectal cancer, given his/her risk factors and presenting symptoms/signs; in other words, how many people out of 100 with the same risk factors and presenting symptoms/signs are likely to have colorectal cancer. A study that validated the algorithm on another large cohort of patients, a proportion of whom had colorectal cancer, found that the algorithm performed very well: it discriminated correctly between cancer and non-cancer patients approximately 90% of the time (i.e. produced higher risk estimates for cancer than non-cancer patients).

We piloted the information to ensure that participants understood it and trusted the algorithm (see the Piloting subsection).

To prevent fatigue, the vignettes were divided into two sets of 10 (sets A and B) and were presented on two different days. Sets were similar in terms of their risk profile (Set A 0.69–57.23%, Median = 4.2%, *M* = 14.0%, *SD* = 19.4%; set B 0.58–56.65%, median = 4.2%, *M* = 14.2%, *SD* = 19.6%). Three practice vignettes were provided at the start of the experiment to help participants calibrate their risk estimates; they represented three levels of risk: low (1%), medium (6%) and high (40%).

All materials (vignettes and questions) were presented online in the form of a questionnaire using the Qualtrics platform. We created four versions of the questionnaire by crossing set order (set A seen first or set B seen first) by information condition (provided or not provided). Each questionnaire version had a different link. Every eligible GP who completed the expression-of-interest form was sent one of the four links, ensuring an equal number of participants for each of the questionnaire versions.

After reading information about the study and agreeing to a statement of consent online, participants completed their demographics and rated their confidence in assessing patients with symptoms that might indicate cancer. They then answered questions about their awareness, availability and use in their clinical practice of cancer risk algorithms and their general feelings towards them—see Kostopoulou et al., (2022, p. 3) for the exact questions participants were asked.^4^ The algorithm information group was then presented with the algorithm information and were asked to indicate their understanding and level of trust in the algorithm. Next, all GPs were presented with the three practice vignettes in a random order and were informed that they were for familiarisation purposes and no data were being collected. During practice, participants in the algorithm information group had access to the algorithm information at wish; by moving the cursor to a specific position on the screen, the algorithm information appeared. In all other aspects, the procedure was identical to the rest of the vignettes that were used for data collection (see below).

**Fig. 2:**
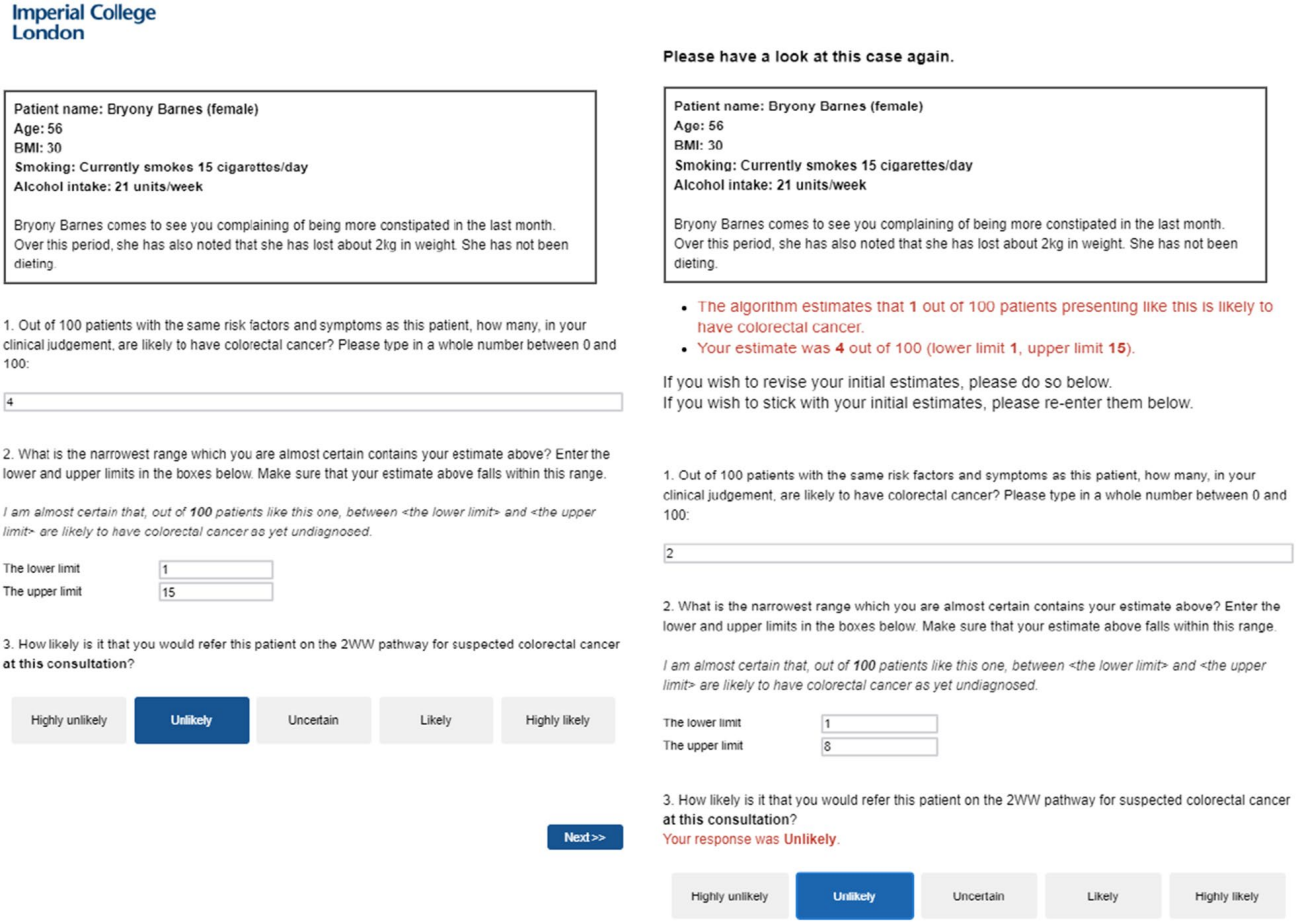
Screenshots of the two consecutive pages of an example vignette showing the vignette, the questions asked before the algorithm was presented (left panel) and the questions and reminders (in red) when the algorithm was presented (right panel). Figure available under a CC-BY4.0 license at https://osf.io/t7gye/

Once they completed practice, participants were presented with ten vignettes from either set A or set B. For each vignette, participants provided (see Fig. 2, left panel):

1. their initial risk estimate as an integer: “Out of 100 patients with the same risk factors and symptoms as this patient, how many, in your clinical judgement, are likely to have colorectal cancer?”;
2. the confidence interval around their risk estimate as two integers: “What is the narrowest range which you are almost certain contains your estimate above?”;
3. and their referral decision: “How likely is it that you would refer this patient on the 2WW pathway for suspected cancer at this consultation?” (5-category response scale from ‘highly unlikely’ to ‘highly likely’)^5^

Next, the same vignette was presented again, and participants were given the algorithm’s risk estimate as an integer out of 100. They were then reminded of their initial responses and asked if they wished to revise them. If participants preferred to stick with their initial responses, they had to type them in again (Fig. 2, right panel). This was done in order to avoid participants not revising their initial responses out of ‘laziness’. After participants completed the ten vignettes of the first session, they were invited to leave feedback on any aspect of the study, if they wished. They were then thanked and informed that a link to the second session would be sent to them in 24 h.

A link to the second session was sent to participants automatically 24 h after completing the first session. At the start of the second session, GPs in the algorithm information group were presented with the algorithm information again. The second session included the set of vignettes not seen in the first session (either set A or set B). Vignettes were presented in a random order, and the procedure was identical to the first session. After completing the ten vignettes of the second session, GPs completed an algorithm disposition questionnaire that gauged the participants’ opinions, attitudes, and emotional responses towards the study algorithm (see Kostopoulou et al., (2022, p. 3) for the seven items of the questionnaire).^6^ Following completion of both sets, GPs were reimbursed £60 for their participation and were sent a certificate of study completion, as well as detailed feedback. The feedback consisted of the 23 vignettes, the QCancer risk estimates, and the participants’ responses.

### Piloting

We piloted the materials with a special focus on the vignettes, the format for eliciting risk estimates and confidence intervals, and the algorithm information. Piloting was conducted in four stages. First, two GPs completed 30 vignettes in two sessions (15 vignettes per session), with different response format for the risk estimates in each session (GPs responded by either using a slider or by entering an integer in a textbox). GPs complained about the repetitiveness of the task; therefore, at the second piloting stage, we reduced the number of vignettes to 20 (10 per session). Three GPs participated in the second piloting stage and used both response formats. They all found the textbox more intuitive than the slider; therefore, we decided to adopt this mode of responding. Based on their feedback, we also added 3 practice cases at the start, to familiarise participants with the task. At the third piloting stage, six GPs read an earlier version of the algorithm information and responded to questions about its clarity and perceived usefulness. As some GPs wished to know more about how the algorithm was validated, we included the relevant information in the description. At the final stage of piloting, 11 GPs (four in the algorithm information group) completed two sessions that closely followed the procedure and materials of the final study. GPs left only positive comments regarding the vignettes in all the pilot studies, they found the vignettes realistic, clear, and engaging.

### Analyses

We ran three multilevel linear regressions with random intercept by GP to investigate the primary and secondary preregistered questions. We also conducted non-preregistered, multilevel linear regressions to either supplement the analysis of the primary questions with additional hypothesis-testing, or to investigate exploratory questions. See the Results section for details about the variables and the models of the confirmatory and exploratory analyses. We used significance testing with the traditional *p*-value threshold of 0.05 on the regression slopes. We reported 95% CIs along with the regression coefficients. We ran the analyses in R (version 4.0.3), and we reproduced them in Stata (version 13.1).

When a statistical test was non-significant, we also assessed the strength of evidence for and against the hypothesis by calculating the Bayes factor (BF; Rouder et al., 2009). We used the Bayes factor calculator of Dienes and Mclatchie (2018). To draw conclusions about the null and alternative hypotheses based on the BFs, we used the traditional thresholds of good enough evidence: 3 for the alternative hypothesis and 1/3 for the null hypothesis (Jeffreys, 1939). In order to calculate the BF, one needs to specify the priors (i.e. predictions of the compared hypotheses). We modelled the priors for the null hypothesis with a point-null model and for the alternative hypotheses with half-normal distributions that had a mode of zero and a SD that depended on the expected effect size. We had directional hypotheses and used the normal distribution rather than the uniform or the heavy-tailed Cauchy, assuming that small effect sizes were more likely than large effect sizes. To identify the SD of the models of the alternative hypotheses, we used the room-to-move heuristic, which is ideal for dichotomous predictors such as the information condition (Dienes, 2019). This heuristic can be used to identify the maximum expected effect size under an alternative hypothesis by considering the constraints of the applied measurement scales (e.g. the difference between a baseline and an experimental group cannot be larger than the difference between the baseline group and the maximum value on the scale with which we measured our outcome variable). Since two SDs distance from the mode of a normal distribution is a good approximation of its plausible maximum effect, we set the SDs of our models at maximum expected effect/2.

We reported all Bayes factors as BF_H(0, SD)_ where H indicates that the prior distribution of the alternative hypothesis is a half-normal distribution, 0 indicates that the mode of the prior distribution is zero, and the SD highlights the SD of the prior distribution (which is also the expected effect size). Since the predictions of the hypotheses can be modelled in multiple ways, we reported robustness regions (Dienes, 2019) along the Bayes factors to assess the robustness of the conclusions based on the chosen SDs of the prior distributions. We reported these robustness regions as RR_Conclusion of the BF_ [min, max], in which min indicates the smallest and max indicates the largest SD of the model with which we would come to the same conclusion.

## Results

We collected 3140 responses from 157 GPs (84 females—54%, *M*_age_ = 43.99 years, *SD*_age_ = 8.74, *M*_experience_ = 14.12 years, *SD*_experience_ = 9.17). Half of the participants (80/157, 51%) were presented with information about the algorithm.

### Feedback about the vignettes

To assess whether GPs found the vignettes engaging and realistic, we analysed their feedback left at the end of the first and second sessions. 21 GPs wished to receive the results of one or more investigations about the hypothetical patients. Some GPs commented on the overall realism of the vignettes and only one of them claimed the cases to be unrealistic, whereas 12 GPs reported that the cases were engaging and/or realistic. For example:

GP 62567: *“The cases presented are typical of patients encountered in general practice.”*

GP 71122: *“Good case scenarios, true to life, with a little more detail than in previous similar studies.”*

### Risk estimates

Following our preregistration, we checked whether any respondents updated their risk estimates more than 6 times (the third of the vignettes) in the opposite direction to the algorithm (Diff_(InitialEst − Algorithm)_ < Diff_(FinalEst − Algorithm)_). This did not happen. Only in 9 responses (0.3% of the total responses) and in no more than 6 responses for the same participant did we observe estimate updating away from the algorithm; hence we did not exclude any participants.

**Fig. 3:**
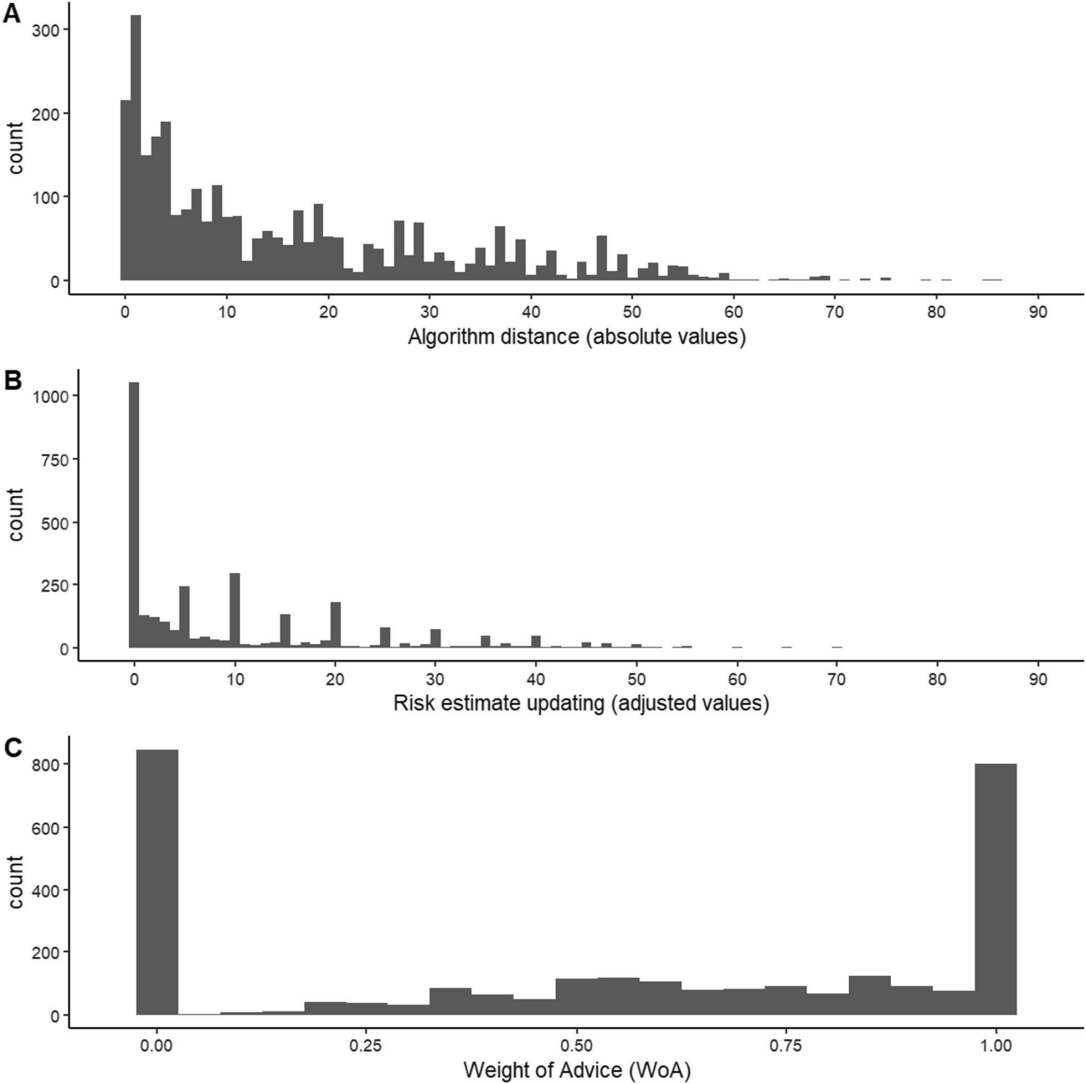
**A** depicts the distribution of the absolute values of algorithm distance (|Diff_(InitialEst − Algorithm)_|), **B** depicts the distribution of the adjusted values of risk estimate updating (adjusted Diff_(InitialEst − FinalEst)_ means that positive values denote updating towards the algorithm), and **C** depicts the distribution of the WoA scores. Figure available under a CC-BY4.0 license at https://osf.io/t7gye/

Figure 3A presents the distributions of the absolute values of algorithm distance (|Diff_(InitialEst − Algorithm)_|). An empty multilevel model with random intercept by GP found that |Diff_(InitialEst − Algorithm)_| was 16.50% on average (*b* = 16.50% [15.58, 17.42], *p* < 0.001).^7^ We used the absolute values of algorithm distance in order to avoid situations where the initial overestimation and underestimation of risk cancelled each other out. In other words, if there were an equal number of cases where InitialEst > Algorithm and cases where InitialEst < Algorithm, then the average algorithm distance would be deceptively small.

Risk estimate updating was denoted as the difference between initial and final risk estimates (Diff_(InitialEst − FinalEst)_). To avoid situations where differences cancelled each other out, we adjusted this variable, so that positive values denoted updating towards the algorithm and negative values denoted updating away from the algorithm. In separate analyses (not preregistered), we used the raw values of estimate updating, with results comparable to those presented here (see Additional file 1). Figure 3B presents the adjusted values of estimate updating. An empty multilevel model indicated that estimate updating (the adjusted variable) was significant (*b* = 10.23% [9.45, 11.01], *p* < 0.001): GPs updated their risk estimates towards the algorithm by 10.23% on average. Moreover, an empty multilevel model found that |Diff_(FinalEst − Algorithm)_| was 6.42% on average (*b* = 6.42% [5.48, 7.36], *p* < 0.001), indicating that the final estimates were not completely in line with the algorithm.

We calculated the weight of advice score (WoA, Harvey & Fischer, 1997) for all responses using the formula:

$$WoA = \frac{{{\text{initial }}\,{\text{risk}}\,{\text{estimate}}\, - \,{\text{final}}\,{\text{ risk}}\,{\text{ estimate}}}}{{{\text{initial}}\,{\text{risk}}\,{\text{estimate}}\, - \,{\text{algorithm}}}}$$

WoA is a percentage shift measure that indicates the extent to which the final risk estimate takes into account both the initial risk estimate and the algorithm. Following prior studies that reported WoA, we replaced all scores larger than 1 with 1, and all negative scores with 0 (we replaced 3% of the responses, 106/3140; e.g. Soll & Larrick, 2009; Schultze et al., 2015; Logg et al., 2019).^8^ We ran an empty multilevel model with random intercept by GP and WoA as the dependent variable. For this analysis, we excluded responses where the initial estimate was the same as the algorithm (WoA cannot be calculated if the denominator is zero; 7% of the responses, 215/3140). The mean value of WoA was 0.54 (*SD* = 0.41, *Median* = 0.60) and the regression model confirmed that it was significantly larger than 0 (*b* = 0.54 [0.50, 0.59], *p* < 0.001). This means that, on average, estimates were updated by over half of the algorithm distance (WoA was also significantly larger than 0.5, *p* = 0.040). However, the distribution of the WoA scores shows that the final risk estimates were rarely positioned in the middle of the algorithm distance (Fig. 2C; 0.4 < WoA < 0.6 in 12% of the responses). In fact, GPs did not update their estimates at all in 29.0% of responses (WoA score = 0), and when they did update them, their final estimates were close to the algorithm (WoA = 1 in 27.2.% of responses, and WoA > 0.8 in 38.2% of responses).

**Fig. 4:**
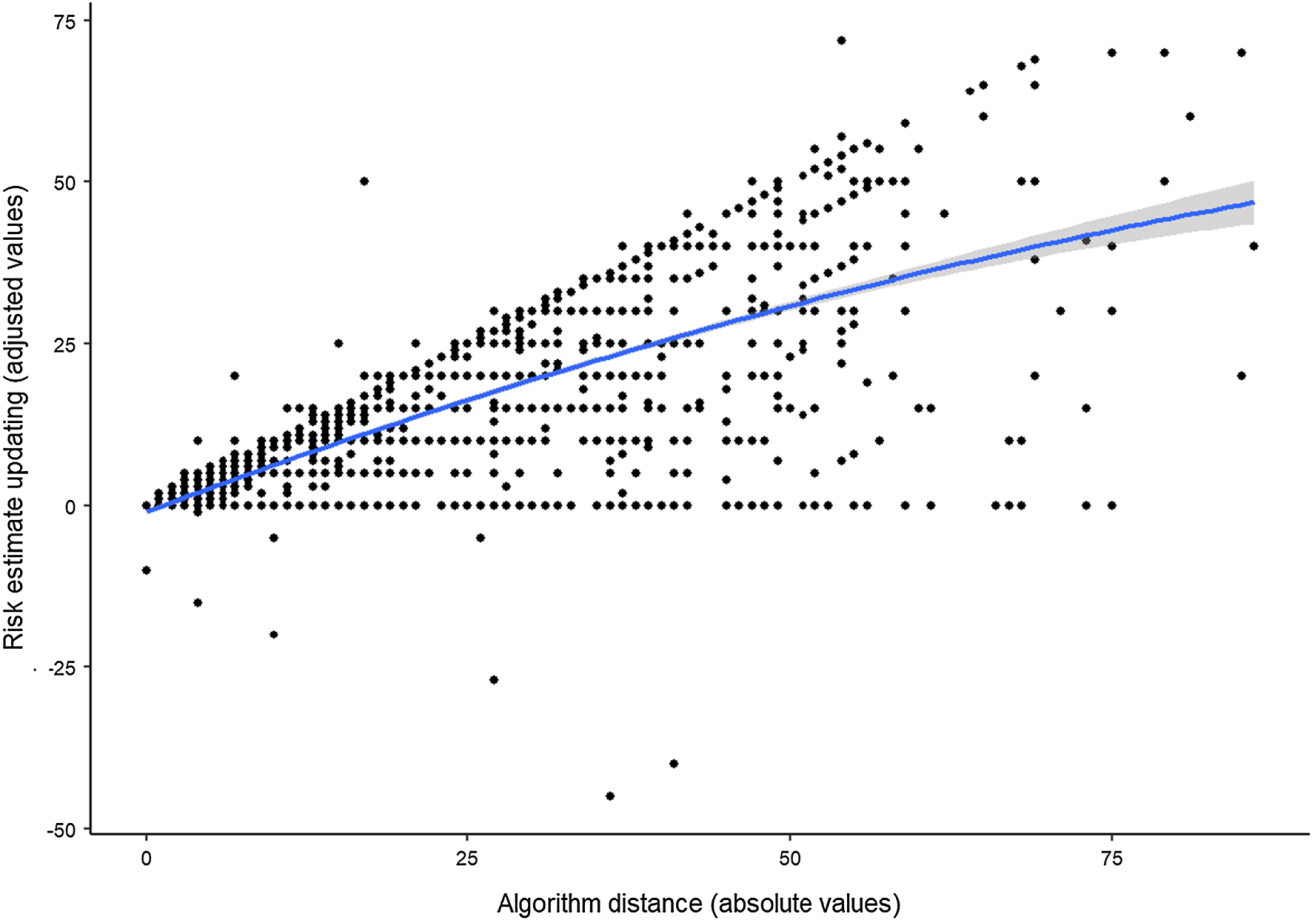
Scatterplot depicting the relationship between the absolute values of algorithm distance (|Diff_(InitialEst − Algorithm)_|) and the adjusted values of estimate updating (adjusted Diff_(InitialEst − FinalEst)_). The blue line represents the quadratic model with the 95% CIs (the grey area around the blue line). Figure available under a CC-BY4.0 license at https://osf.io/t7gye/

Next, we tested (1) whether estimate updating was associated with algorithm distance, (2) whether this association was linear or quadratic, and (3) whether estimate updating was influenced by having information about the algorithm. To test our preregistered questions, we constructed a multiple multilevel regression model with random intercept by GP, where we regressed the adjusted Diff_(InitialEst − FinalEst)_ on |Diff_(InitialEst − Algorithm)_| as a linear predictor, |Diff_(InitialEst − Algorithm)_| as a quadratic predictor, and the information condition. Both the linear (*b*_1_ = 0.78% [0.74, 0.83], *p* < 0.001) and quadratic terms (*b*_2_ = − 0.002% [− 0.003, − 0.001], *p* < 0.001) were significant (see Fig. 4 for the visualisation of the quadratic trend). The coefficient of the linear term is the instantaneous change or the slope at the specific point where Diff_(InitialEst − Algorithm)_ = 0. The value of the coefficient (*b*_1_ = 0.78) implies that a small difference has a big impact. The coefficient of the quadratic term (*b*_2_ = − 0.002) measures how the slope changes as algorithm distance increases. Its negative value suggests that the large initial slope (*b*_1_ = 0.78) decreases as algorithm distance increases. That is, the effect of going from a value of 0 to 1 has a bigger impact than, say, going from a value of 5 to 6. This suggests that the change is decelerating. Since the coefficient of the quadratic term is small (*b*_2_ = − 0.002), it does not turn the curve over (inverted-U) within the range of the existing algorithm distance values (see Fig. 4.).

The presence of the quadratic term means that there are several different slopes; a relatively large slope for an algorithm distance of 0 and smaller slopes for bigger algorithm distances. The weighted average of all these slopes is 0.67 (*b* = 0.67% [0.65, 0.69], *p* < 0.001). This is the coefficient of the linear term, when we exclude the quadratic term from the regression. On average, a one-unit increase in algorithm distance produces an increase of 0.67 in estimate updating. The presence of the quadratic term suggests, however, that this simple average is misleading because the size of the slope varies for different algorithm distances.

We detected no significant difference in risk estimate updating between information conditions (*b* = 0.37% [− 1.17, 1.90], *p* = 0.643). Indeed, the Bayes factor indicated good enough evidence in favour of the null hypothesis for the information condition (BF_H(0,3.5)_ = 0.326, RR_BF<3_[3.5, Inf]). When we regressed risk estimate updating on information condition alone, the relationship was not significant (*b* = 1.42 [− 0.12, 2.96], *p* = 0.07), though there was weak Bayesian evidence towards more updating when algorithm information was provided, in accordance with the hypothesis (BF_H(0,3.5)_ = 1.95, RR_BF<3_[1.7, 22.6]; NB. this analysis was not preregistered).

### Confidence intervals for risk estimates

We calculated the confidence intervals for the initial and final risk estimates by subtracting the lower limits from the upper limits of each confidence interval. We excluded responses where the confidence interval of either the initial or the final risk estimate was smaller than zero (38/3140, 1.21%). The mean width of the confidence intervals for the initial estimates was 18.48 (*SD* = 15.71), and for the final estimates 16.04 (*SD* = 16.14). The mean value for confidence interval updating (Diff_CiInitial − CiFinal_) was 2.44 (*SD* = 12.59).

First, we tested whether confidence interval updating differed from zero. We created an empty multilevel regression model with Diff_CiInitial − CiFinal_ as the dependent variable and a random intercept by GP. Although GPs did not update their confidence intervals in 42% of the responses (1296/3112), confidence interval width significantly reduced after the algorithm was provided (*b* = 2.26 [1.64, 2.87], *p* < 0.001). Note that GPs widened their confidence intervals in 17% (527/3112) and narrowed them in 41% (1289/3112) of the responses. Next, we investigated whether confidence interval updating is related to algorithm distance, so we extended the above model with |Diff_(InitialEst − Algorithm)_| as a linear predictor. We found a significant negative relationship (*b* = − 0.06 [− 0.08, − 0.03], *p* < 0.001): the smaller the algorithm distance was, the more GPs increased their confidence in their estimate. (NB. these analyses were not preregistered).

Finally, to test whether one`s confidence in their initial estimate negatively impacts the utilisation of advice we regressed risk estimate updating (adjusted variable) on the confidence interval of the initial risk estimate. The results revealed a positive relationship (*b* = 0.32 [0.29, 0.36], *p* < 0.001): the more certain GPs were about their initial risk estimate (the narrower the confidence intervals), the less they updated their risk estimate after seeing the algorithm.

## Discussion

Research on advice taking concurs that humans systematically overweigh their own judgement compared to human and algorithmic advice. This study aimed to investigate the generalisability of the advice discounting phenomenon to experts performing an important task using the judge-advisor system and evidence-based algorithmic advice. We also examined whether the influence of algorithmic advice depends on algorithm distance and whether providing information about the derivation and accuracy of the algorithm improves its utilisation.

### Advice discounting

Our primary analysis revealed that, on average, GPs placed their final estimates closer to the advice than to their own initial estimate, resulting in an average WoA score of 0.54, which is one of the highest in the literature. This high level of compliance with the advice is not in line with the egocentric advice discounting phenomenon, namely, that judges generally adhere to their initial estimate, and they adjust it by only a “token” amount (Yaniv & Kleinberger, 2000). The average WoA score implies that GPs considered their own estimates and the algorithmic advice to be almost equally valid.

Nonetheless, a closer look at the distribution of the WoA scores reveals that GPs’ estimate updating behaviour cannot be fully described with a simple averaging strategy (i.e. “take the average of the initial estimate and advice”). In over half of the responses, GPs either completely ignored (29.0% of the responses) or entirely accepted (27.2% of the responses) the offered advice, and they placed their final estimate close to the middle of the algorithm distance only in 12% of the responses. These results suggest that GPs regularly employed at least two strategies: 1) choosing either the initial estimate or the advice and 2) averaging the two values. This finding is consistent with previous research on advice taking (Soll & Larrick, 2009; Soll & Mannes, 2011). Interestingly, GPs in our study chose the advice two to three times more often than the judges in previous studies, who typically chose the advice less than 10% of the time (cf., Soll & Larrick, 2009; Soll & Mannes, 2011). Furthermore, GPs seemed to average their initial estimate and the advice roughly half of the time compared to judges in previous studies (in the studies of Soll and Larrick [2009], and Soll and Mannes [2011], 20% of responses had typically a WoA score between 0.4 and 0.6]). The difference in the tendency to choose the advice may be attributable to the fact that advice, in our study, came from an evidence-based algorithm rather than from human judges. Indeed, some experimental evidence supports that algorithmic advice tends to elicit substantially higher WoA scores than human advice (Logg et al., 2019). However, the extent to which human and algorithmic advice are utilised should not necessarily be compared to the same standard. An abundant number of studies demonstrated that pure averaging (a WoA score of 0.5) is the optimal strategy for human advice, assuming that the judge and the advisor are from the same population (Dawes & Corrigan, 1974; Einhorn et al., 1977). In contrast, when it comes to the algorithm employed in our study, the level of advice utilisation that leads to an optimal performance is likely to be close to a WoA score of 1, implying that there remains substantial room for improvement and that WoA scores above 0.5 can still be interpreted as evidence for advice discounting.

### Advice distance effect

The advice distance effect, the negative relationship between advice distance and WoA, has been interpreted in the literature as a manifestation of egocentric advice discounting. However, it was suggested that a simple stimulus–response model could also account for this phenomenon (Schultze et al., 2015). To distinguish between these two models, we tested the strength of the relationship between advice distance and risk estimate updating rather than WoA. We replicated the advice distance effect by finding a negative quadratic relationship; the proportion of estimate updating reduced as advice distance enlarged. Nonetheless, the quadratic relationship was weak, and estimate updating increased in a monotone fashion for the whole range of the observed algorithm distances. Therefore, the stimulus–response model fits our data better than egocentric advice discounting, which would predict a reversal in estimate updating as advice distance grows. Although our finding is in stark contrast with the results of early studies of the phenomenon (e.g. Minson et al., 2011; Yaniv, 2004a, 2004b; Yaniv and Milyavsky, 2007), it is not unprecedented. A recent study using general knowledge tasks across six experiments found no evidence of a strong negative quadratic relationship between advice distance and estimate updating (Schultze et al., 2015). In fact, there was evidence for a quadratic relationship in only one of the experiments, and a positive linear model turned out to be the best fit in the remaining experiments. This finding proved to be independent of the communicated quality of advice, and the perceived distance from the advice (i.e. whether the judges perceived the advice as dissimilar or similar to their own judgement). Nonetheless, advice was not real in any of these experiments, since it was designed to match predefined advice distance values based on the initial estimates of the judges. Our findings extend these results by demonstrating that they generalise to tasks of high importance with real and high-quality advice.

### Information about the algorithm

We found evidence that providing information about the derivation and accuracy of an algorithm has no effect on estimate updating. We expected to find stronger algorithm uptake when information was provided based on earlier studies demonstrating that judges put more weight on advice that comes from advisors with good past performance and high credibility than advisors with no such credentials (Yaniv, 2004b). Our finding is surprising given the fact that after reading the presented algorithm information most GPs reported that the information was understandable and trustworthy, and that they would like to have an algorithm like this in their own practice (see Kostopoulou et al., 2022, pp. 4–5). Nonetheless, we could not ask GPs in the no-information condition these questions, so it was not possible to test whether we improved trust in the algorithm by presenting the algorithm information. It may be the case that GPs have a sufficient understanding of how and how well cancer risk algorithms work and they have a high level of trust in them, which could undermine the impact of the algorithm information intervention. In fact, at the beginning of the study, the majority of GPs reported positive attitudes towards cancer risk algorithms in general (Kostopoulou et al., 2022, p. 4). Hence, it is possible that our algorithm information merely confirmed the existing positive beliefs of the GPs, but it could not further elevate them. More research is needed to test whether the impact of algorithm information on algorithm uptake is moderated by judges` attitudes towards algorithms in general.

### The link between confidence and advice taking

Confidence in one’s own judgement has been identified as a reliable predictor of advice utilisation. Namely, low confidence compared to high confidence is associated with an increased reliance on advice (e.g. Gino et al., 2012; Harvey & Fischer, 1997; Hütter & Fiedler, 2019; See et al., 2011). We tested this relationship in a preregistered analysis using a response level confidence measure that gauged uncertainty in the initial and final risk estimates separately (i.e. the confidence interval measure). As expected, we found a positive relationship between the width of initial confidence intervals and subsequent estimate updating. This result is also in harmony with the overconfidence account of advice discounting, which is built on the assumption that confidence negatively affects advice taking.

Confidence is not only interesting as a predictor of estimate updating; it is relevant in its own right, as it allows us to understand better the impact of advice distance. Recent research focusing on the link between advice distance and confidence updating revealed a monotone, negative relationship (Hutter & Ache, 2016; Moussaid et al., 2013; Schultze et al., 2015). This relationship is not unexpected; models of confidence in advice taking emphasise the role of the perceived agreement or consensus between the judge and the advisor in confidence updating, and they assume a negative link between them (e.g. Budescu, 2006; Yaniv et al., 2009). Our exploratory analysis using the response level confidence measure revealed evidence for the expected negative relationship suggesting that GPs felt more validated by the algorithm when it was close to rather than distant from their initial estimate. Nonetheless, the relationship was not sufficiently strong to overpower the overall increase in confidence after the algorithm and so the regression model predicts increase in confidence for large algorithm distance values as well (predicted confidence change is 0 for algorithm distance of 56.1) implying that GPs in general found the algorithm to be in line with their own judgements.

### Limitations

Algorithmic computerised decision support systems are increasingly growing in number to support clinical judgments and decisions (Sutton et al., 2020). These tools are typically used to help the diagnosis of patients, but the advice and recommendations generated by them can come in various forms. For instance, since first impressions strongly impact the subsequent diagnostic process (Kostopoulou et al., 2017b), a tool that offers a list of diagnostic alternatives (“differential diagnoses”) based on the available patient data can aid GPs in making the correct diagnosis (Kostopoulou et al., 2017a). This type of differential diagnoses generators can also provide probabilities for each diagnostic suggestion, calculated from probabilistic algorithms. In this study, we focused on a cancer risk algorithm, the QCancer (Hippisley-Cox & Coupland, 2013a, 2013b), as a single decision aid, and asked participants specifically to consider the risk of cancer. Therefore, the generalisability of our conclusions and practical implications is limited to this type of risk calculators.

To operationalise clinical judgements, we used clinical vignettes as they offer a good balance between experimental control and generalisability to clinical settings. We aimed to make them realistic by conveying most of the information in a narrative format, while keeping them as brief as possible to retain sufficient experimental control and keep the level of noise in measurement low. Nonetheless, our vignettes may still appear artificial in comparison to everyday clinical practice, which is a common critique of vignette-based studies (e.g. Spalding & Phillips, 2007). For instance, GPs typically meet their patients more than once and can interact with them which provides the GPs with rich clinical and contextual information about the patients and their problem. GPs can also order additional test results to reduce uncertainty regarding their risk assessment (this was raised by some of our participants). However, these discrepancies do not necessarily undermine the validity of our conclusions, as our conclusions are not conditional on the vignettes perfectly recreating real-life clinical settings. Vignette based studies can provide valid conclusions by simply approximating real-life settings as approximation is typically sufficient to simulate the cognitive processes used in the real-life settings (e.g. Evans et al., 2015). Importantly, the participants of our pilot studies and of the main study reported the vignettes to be mostly realistic supporting the assumption that our vignettes managed to simulate real-life cognitive processes used in the assessment of cancer risk.

The judge-advisor system allowed us to investigate the influence of algorithmic advice on clinical judgements in a pre–post-design. However, this design may not be a good approximation of the everyday practice of GPs, who are not required to form initial risk estimates when they assess patients. Nonetheless, in the absence of an initial estimate, we expect that judges put their final estimate closer to the algorithm than when they need to form an explicit initial estimate (cf., anchoring effect by Tversky & Kahneman, 1974). Hence, the potential bias introduced by the experimental design is more likely to have deflated rather than inflated the influence of algorithmic advice.

Finally, GPs invited for this study were informed that the study is about algorithms, so it is possible that the participating GPs were more interested in algorithms and more open to revising their professional judgements and decisions in light of algorithmic advice than others (i.e. selection bias). Moreover, the application of a pre–post-design may have evoked comparative performance anxiety in some of the GPs as their initial estimates could be directly compared to the algorithm. This effect may have further inflated the level of agreement with the algorithm by compelling GPs to accept the estimate of the algorithm regardless of the extent of algorithm distance. Also, this effect may have diminished the impact of algorithm information on risk updating by creating a ceiling effect. Future research is needed to explore these possibilities.^9^

### Practical implications

Cancer risk algorithms play an essential role in the plans of NHS England, which aims to elevate the level of stage 1 and 2 cancer diagnoses from 55 to 75% over a ten-year period (Cancer Research UK, 2021; NHS Digital, 2021). Although the established hypothesis of egocentric advice discounting would predict that algorithms will fail to make a difference (e.g. Yaniv, 2004a, 2004b), our findings suggest that GPs frequently change their judgements when they have access to an algorithm highlighting their potential to improve the early detection of cancer. We suspect that this is due to the fact that the provided algorithm was evidence-based, and that GPs had a generally positive view of risk algorithms in clinical practice. If this is the case, then any risk calculator or evidence-based algorithm that estimates an uncertain quantity has good prospects of improving human judgements, as long as users have a positive view of the algorithm. One may wonder whether this cooperative attitude can be retained in the long-term when GPs use an algorithm in their clinical practice. For instance, algorithm aversion postulates that judges quickly lose trust in an algorithm when they see it make mistakes, even if the algorithm is more accurate on average than the judge (Dietvorst & Bharti, 2020; Dietvorst et al., 2015). Nevertheless, GPs do not see large numbers of patients with a possible cancer and do not receive feedback on their cancer status at such large numbers to enable them to estimate the accuracy of the algorithm. To evaluate the accuracy of a probabilistic algorithm, GPs would need to rely on clinic-level data assessing whether the deployment of the algorithm in the clinic as a whole (or in a group of clinics) has increased the number of cancers detected over a period of time, while reducing the number of false positives (Kostopoulou et al., 2020). Therefore, we do not expect that algorithm aversion, as defined in the literature, imposes a threat on the uptake of risk algorithms in clinical practice.

Our findings have another important and positive implication by challenging the egocentric advice distance hypothesis that claims that judges reject or do not seriously consider advice that conflicts with their own judgement. Advice carries the highest value when it has the prospect of overturning a poor judgement or decision, which is especially important in the context of cancer risk estimation. Our study implies that GPs are willing to revise their initial judgement regarding the risk of cancer in the face of a conflicting piece of advice coming from an algorithm (cf. Chiang et al., 2015), an action that is critical to the improvement of the early diagnosis of cancer and cancer outcomes in general. The fact that the pattern of results in our study matches well that of an earlier study using general knowledge tasks (Schultze et al., 2015) makes it likely that the findings generalise well to a series of estimation problems. Nonetheless, future studies should test whether judges accept conflicting advice from an algorithm, when it comes to the diagnosis of a different cancer type, to the assessment of a non-life-threatening disease, or to problems outside primary care or the clinical context in general, such as financial or judicial judgements.

## Conclusion

This study tested how well the advice discounting phenomenon generalises when experts are making real-life judgements with the help of an evidence-based algorithm. We found that GPs placed their final estimates closer to the algorithm than to their initial estimates, challenging the idea that judges strongly adhere to their own opinion. This level of compliance with advice is one of the highest in the literature. Yet, advice discounting was still apparent, as advice from evidence-based algorithms should be accepted to a much higher degree. Unlike earlier studies with human advice, providing information about the algorithm did not improve reliance on advice, hence, future research should aim to identify more effective ways to influence advice taking. We also found that the influence of advice did not diminish substantially when a conflict emerged between a GP’s estimate and the algorithm, implying that algorithmic advice carries great potential to influence clinical judgements.

## Supplementary Information

**Additional file 1.** Supplementary Materials.

## Data Availability

The materials are provided in the online version of the paper by Kostopoulou et al., (2022) (https://www.nature.com/articles/s43856-021-00069-1). The anonymised data and the analysis script can be accessed at https://osf.io/7e2mv (OSF project link: https://osf.io/bpd24/).

## Footnotes

1 WoA was not reported in the paper, only the average difference between the initial estimates and the advice (139.58 units), and the final estimates and the advice (104.67). We calculated the WoA by dividing these scores.

2 Confidence of the advisor is another influential factor (Sniezek & Van Swol, 2001; Van Swol & Sniezek, 2005); however, confidence is unlikely to bear relevance to algorithmic advice taking as algorithmic advice frequently comes in a form of a single point estimate.

3 We opted to use the interval format rather than a Likert scale of subjective confidence to maximise the validity of our measure.

4 Analyses of these variables are reported by Kostopoulou et al. (2022).

5 For the analysis of this variable, see Kostopoulou et al. (2022).

6 For the analysis of this variable, see Kostopoulou et al. (2022).

7 NB. In accordance with the preregistration, we ran all multilevel regression analyses with random intercept by GP. In the Additional file 1, we reported the same analyses with random intercepts by GP and vignette.

8 NB. This step was not preregistered, so we analysed the non-trimmed data as well. The pattern of results and the conclusions match across the trimmed and non-trimmed data.

9 We thank an anonymous reviewer for raising this point.

## References

[CR1] Birnbaum MH, Stegner SE (1979). Source credibility in social judgment: Bias, expertise, and the judge's point of view. Journal of Personality and Social Psychology.

[CR2] Block RA, Harper DR (1991). Overconfidence in estimation: Testing the anchoring-and-adjustment hypothesis. Organizational Behavior and Human Decision Processes.

[CR3] Budescu, D. V. (2006). Confidence in aggregation of opinions from multiple sources. *Information sampling and adaptive cognition*, 327–352.

[CR4] Chiang PP, Glance D, Walker J, Walter FM, Emery JD (2015). Implementing a QCancer risk tool into general practice consultations: An exploratory study using simulated consultations with Australian general practitioners. British Journal of Cancer.

[CR5] Cancer Research UK. (2021) *Early detection and diagnosis of cancer: A roadmap to the future*. https://www.cancerresearchuk.org/sites/default/files/early_detection_diagnosis_of_cancer_roadmap.pdf.

[CR6] Dawes RM, Corrigan B (1974). Linear models in decision making. Psychological Bulletin.

[CR7] Dienes Z (2019). How do I know what my theory predicts?. Advances in Methods and Practices in Psychological Science.

[CR8] Dienes Z, Mclatchie N (2018). Four reasons to prefer Bayesian analyses over significance testing. Psychonomic Bulletin & Review.

[CR9] Dietvorst BJ, Bharti S (2020). People reject algorithms in uncertain decision domains because they have diminishing sensitivity to forecasting error. Psychological Science.

[CR10] Dietvorst BJ, Simmons JP, Massey C (2015). Algorithm aversion: People erroneously avoid algorithms after seeing them err. Journal of Experimental Psychology: General.

[CR11] Einhorn HJ, Hogarth RM, Klempner E (1977). Quality of group judgment. Psychological Bulletin.

[CR12] Evans SC, Roberts MC, Keeley JW, Blossom JB, Amaro CM, Garcia AM, Stough CO, Canter KS, Robles R, Reed GM (2015). Vignette methodologies for studying clinicians’ decision-making: Validity, utility, and application in ICD-11 field studies. International Journal of Clinical and Health Psychology.

[CR13] Freedman JL (1964). Involvement, discrepancy, and change. The Journal of Abnormal and Social Psychology.

[CR14] Gandhi TK, Kachalia A, Thomas EJ, Puopolo AL, Yoon C, Brennan TA, Studdert DM (2006). Missed and delayed diagnoses in the ambulatory setting: A study of closed malpractice claims. Annals of Internal Medicine.

[CR15] Gardner PH, Berry DC (1995). The effect of different forms of advice on the control of a simulated complex system. Applied Cognitive Psychology.

[CR16] Gino F, Schweitzer ME (2008). Blinded by anger or feeling the love: How emotions influence advice taking. Journal of Applied Psychology.

[CR17] Gino F, Brooks AW, Schweitzer ME (2012). Anxiety, advice, and the ability to discern: Feeling anxious motivates individuals to seek and use advice. Journal of Personality and Social Psychology.

[CR18] Goodwin P, Fildes R (1999). Judgmental forecasts of time series affected by special events: Does providing a statistical forecast improve accuracy?. Journal of Behavioral Decision Making.

[CR19] Grove WM, Zald DH, Lebow BS, Snitz BE, Nelson C (2000). Clinical versus mechanical prediction: A meta-analysis. Psychological Assessment.

[CR20] Harvey N, Fischer I (1997). Taking advice: Accepting help, improving judgment, and sharing responsibility. Organizational Behavior and Human Decision Processes.

[CR21] Hippisley-Cox J, Coupland C (2013). Symptoms and risk factors to identify men with suspected cancer in primary care: Derivation and validation of an algorithm. British Journal of General Practice.

[CR22] Hippisley-Cox J, Coupland C (2013). Symptoms and risk factors to identify women with suspected cancer in primary care: Derivation and validation of an algorithm. British Journal of General Practice.

[CR23] Hütter M, Ache F (2016). Seeking advice: A sampling approach to advice taking. Judgment & Decision Making.

[CR24] Hütter M, Fiedler K (2019). Advice taking under uncertainty: The impact of genuine advice versus arbitrary anchors on judgment. Journal of Experimental Social Psychology.

[CR25] Insko CA, Murashima F, Saiyadain M (1966). Communicator discrepancy, stimulus ambiguity, and influence. Journal of Personality.

[CR26] Jeffreys H (1939). Theory of probability.

[CR27] Kostopoulou O, Arora K, Palfi B (2022). Using cancer risk algorithms to improve risk estimates and referral decisions. Communications Medicine.

[CR28] Kostopoulou O, Nurek M, Delaney BC (2020). Disentangling the relationship between physician and organizational performance: A signal detection approach. Medical Decision Making.

[CR29] Kostopoulou O, Porat T, Corrigan D, Mahmoud S, Delaney BC (2017). Diagnostic accuracy of GPs when using an early-intervention decision support system: A high-fidelity simulation. British Journal of General Practice.

[CR30] Kostopoulou O, Sirota M, Round T, Samaranayaka S, Delaney BC (2017). The role of physicians' first impressions in the diagnosis of possible cancers without alarm symptoms. Medical Decision Making..

[CR31] Kruger J (1999). Lake Wobegon be gone! The" below-average effect" and the egocentric nature of comparative ability judgments. Journal of Personality and Social Psychology.

[CR32] Lim JS, O'Connor M (1995). Judgemental adjustment of initial forecasts: Its effectiveness and biases. Journal of Behavioral Decision Making.

[CR33] Logg JM, Minson JA, Moore DA (2019). Algorithm appreciation: People prefer algorithmic to human judgment. Organizational Behavior and Human Decision Processes.

[CR34] Meehl, P. E. (1954). *Clinical versus statistical prediction: A theoretical analysis and a review of the evidence.* University of Minnesota Press.

[CR35] Minson JA, Liberman V, Ross L (2011). Two to tango: Effects of collaboration and disagreement on dyadic judgment. Personality and Social Psychology Bulletin.

[CR36] Moussaïd M, Kämmer JE, Analytis PP, Neth H (2013). Social influence and the collective dynamics of opinion formation. PLoS ONE.

[CR37] Neal RD (2009). Do diagnostic delays in cancer matter?. British Journal of Cancer.

[CR38] NHS Digital. (2021). *Case-mix adjusted percentage of cancers diagnosed at stages 1 and 2 by CCG in England, 2019.* https://digital.nhs.uk/data-and-information/publications/statistical/case-mix-adjusted-percentage-cancers-diagnosed-at-stages-1-and-2-by-ccg-in-england/2019.

[CR39] Önkal D, Goodwin P, Thomson M, Gönül S, Pollock A (2009). The relative influence of advice from human experts and statistical methods on forecast adjustments. Journal of Behavioral Decision Making.

[CR40] Rouder JN, Speckman PL, Sun D, Morey RD, Iverson G (2009). Bayesian t tests for accepting and rejecting the null hypothesis. Psychonomic Bulletin & Review.

[CR41] Schultze T, Rakotoarisoa AF, Schulz-Hardt S (2015). Effects of distance between initial estimates and advice on advice utilization. Judgment & Decision Making.

[CR42] See KE, Morrison EW, Rothman NB, Soll JB (2011). The detrimental effects of power on confidence, advice taking, and accuracy. Organizational Behavior and Human Decision Processes.

[CR43] Sherif, M., & Hovland, C. I. (1961). Social judgment: Assimilation and contrast effects in communication and attitude change. 10.1037/h0048480 13474895

[CR44] Sniezek JA, Buckley T (1995). Cueing and cognitive conflict in judge-advisor decision making. Organizational Behavior and Human Decision Processes.

[CR45] Sniezek JA, Schrah GE, Dalal RS (2004). Improving judgement with prepaid expert advice. Journal of Behavioral Decision Making.

[CR46] Sniezek JA, Van Swol LM (2001). Trust, confidence, and expertise in a judge-advisor system. Organizational Behavior and Human Decision Processes.

[CR47] Soll JB, Larrick RP (2009). Strategies for revising judgment: How (and how well) people use others’ opinions. Journal of Experimental Psychology: Learning, Memory, and Cognition.

[CR100] Soll JB, Mannes AE (2011). Judgmental aggregation strategiesdepend on whether the self is involved. International Journal of Forecasting.

[CR48] Spalding NJ, Phillips T (2007). Exploring the use of vignettes: From validity to trustworthiness. Qualitative Health Research.

[CR49] Stevens SS (1957). On the psychophysical law. Psychological Review.

[CR50] Sutton RT, Pincock D, Baumgart DC, Sadowski DC, Fedorak RN, Kroeker KI (2020). An overview of clinical decision support systems: Benefits, risks, and strategies for success. NPJ Digital Medicine.

[CR51] Tversky A, Kahneman D (1974). Judgment under uncertainty: Heuristics and biases. Science.

[CR52] Tversky A, Koehler DJ (1994). Support theory: A nonextensional representation of subjective probability. Psychological Review.

[CR53] Van Swol LM, Sniezek JA (2005). Factors affecting the acceptance of expert advice. British Journal of Social Psychology.

[CR54] Yaniv I (2004). Receiving other people’s advice: Influence and benefit. Organizational Behavior and Human Decision Processes.

[CR55] Yaniv I (2004). The benefit of additional opinions. Current Directions in Psychological Science.

[CR101] Yaniv I, Choshen-Hillel S, Milyavsky M (2009). Spurious consensus and opinion revision: Why might people be more confident intheir less accurate judgments?. Journal of Experimental Psychology: Learning, Memory, and Cognition.

[CR56] Yaniv I, Foster DP (1995). Graininess of judgment under uncertainty: An accuracy-informativeness trade-off. Journal of Experimental Psychology: General.

[CR57] Yaniv I, Foster DP (1997). Precision and accuracy of judgmental estimation. Journal of Behavioral Decision Making.

[CR58] Yaniv I, Kleinberger E (2000). Advice taking in decision making: Egocentric discounting and reputation formation. Organizational Behavior and Human Decision Processes.

[CR59] Yaniv I, Milyavsky M (2007). Using advice from multiple sources to revise and improve judgments. Organizational Behavior and Human Decision Processes.

