# Supplementary material for: Algorithm-based advice taking and clinical judgement: impact of advice distance and algorithm information
Source: Cogn Res Princ Implic. 2022 Jul 27;7:70. doi: 10.1186/s41235-022-00421-6 (PMC9329504; doi:10.1186/s41235-022-00421-6)
Supplement: Supplementary file 1 — Additional file 1. Supplementary Materials. [file 41235_2022_421_MOESM1_ESM.pdf]

Algorithm-based advice taking and clinical judgment: impact of advice distance and algorithm information

Supplementary Materials

Palfi, B.[1], Arora, K.[1], Kostopoulou, O.[1]

[1]Imperial College London, Department of Surgery & Cancer

Robustness of the risk estimate updating analyses

To assess the robustness of the preregistered analyses that used adjusted risk estimate updating as the dependent variable, we repeated these analyses using the raw values of both estimate updating ($Diff_{(InitialEst\ -\ FinalEst)}$) and algorithm distance ($Diff_{(InitialEst\ -\ Algorithm)}$). First, we ran an empty multilevel model with random intercept by GP. We found that estimate updating significantly differed from zero (b = 3.22% [2.57, 3.88], p < .001). Next, we created a multiple, multilevel regression model with algorithm distance as both a linear and a cubic term ($Diff_{(InitialEst\ -\ Algorithm)}^3$), and information condition. We opted for a cubic rather than a quadratic term, because the variable was not restricted to the positive values anymore, and we expected symmetrically curved lines for its positive and negative values. Both the linear term (b = 0.66% [0.65, 0.68], p < .001) and the cubic term were significant (b = -0.00002% [-0.00003, -0.00001], p < .001). Figure S1 shows the cubic relationship between risk estimate updating and algorithm distance. Information condition was a non-significant predictor (b = 0.45% [-0.71, 1.61], p = .451), and the Bayes factor revealed data insensitivity ($BF_{H(0,1.8)}$ = 0.62, $RR_{1/3<BF<3}$[0, 3.6]).

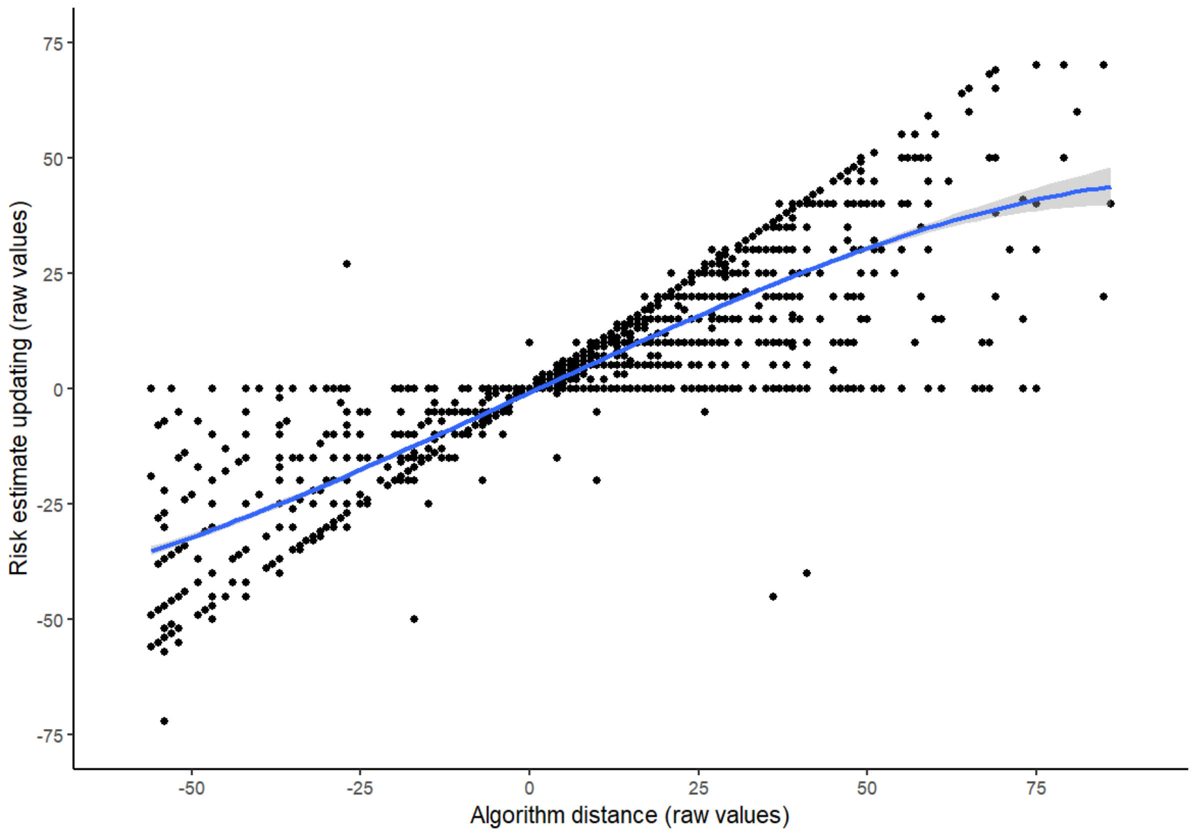

Figure S1. A scatterplot demonstrating the relationship between the raw values of algorithm distance ($Diff_{(InitialEst – Algortihm)}$) and risk estimate updating ($Diff_{(InitialEst – FinalEst)}$). The blue line represents the cubic trend with the 95% CIs (the grey area around the blue line).

Robustness of the regression analyses to the inclusion of a random intercept by vignette

We repeated the multilevel regression analyses reported in the main paper with random intercept by GP and vignette. Table S1 shows the results of the analyses of risk estimates and their updating: $|Diff_{(InitialEst - Algorithm)}|$, WoA and adjusted $Diff_{(InitialEst - FinalEst)}$. Table S2 summarizes the results of the analyses of the confidence intervals for risk estimates with random intercept by GP and vignette.

Table S1.

Summary of the regression models with random intercepts by GP and vignette of the dependent variables measuring risk estimates and their updating. The first four models are empty, i.e., do not include any predictors.

	b	95% CI	p	conclusion
Model: $\lvert Diff_{(InitialEst - Algorithm)} \rvert = \beta_0$				
β_0	16.50%	12.53, 20.47	< .001	unchanged
Model: adjusted $Diff_{(InitialEst - FinalEst)} = \beta_0$ (model failed to converge)				
β_0	10.235	7.43, 13.04	< .001	unchanged
Model: $\lvert Diff_{(FinalEst - Algorithm)} \rvert = \beta_0$				
β_0	6.42%	4.85, 7.99	< .001	unchanged
Model: $WoA = \beta_0$				
β_0 against 0	0.54%	0.49, 0.59	< .001	unchanged
β_0 against 0.5			.094	changed
Model: adjusted $Diff_{(InitialEst - FinalEst)} = \beta_0 + \beta_1 \lvert Diff_{(InitialEst - Algorithm)} \rvert + \beta_2 Diff_{(InitialEst - Algorithm)}^2 + \beta_3 Diff_{(Information\ condition)}$				
β_1	0.78%	0.74, 0.83	< .001	unchanged
β_2	-0.0024%	0.001, 0.003	< .001	unchanged
β_3	0.37%	-1.17, 1.91	.640	unchanged

Table S2.

Summary of the regression models with random intercepts by GP and vignette of the dependent variables measuring confidence intervals for risk estimates. The first model is empty, i.e., does not include any predictors.

	b	95% CI	p	conclusion
Model: $Diff_{CiInitial - CiFinal} = \beta_0$				
β_0	2.25%	0.38, 4.13	= .028	unchanged
Model: $Diff_{CiInitial - CiFinal} = \beta_0 + \beta_1 \lvert Diff_{(InitialEst - Algorithm)} \rvert$				
β_0	0.02	-0.005, 0.045	= .112	changed
Model: adjusted $Diff_{(InitialEst - FinalEst)} = \beta_0 + \beta_1 Ci_{Initial}$				
β_1	0.21	0.18, 0.25	< .001	unchanged
